# Supplementary figures and images for: New data on tail lengths and variation along the caudal series in the non-avialan dinosaurs
Source: PeerJ. 2021 Feb 15;9:e10721. doi: 10.7717/peerj.10721 (PMC7891087; doi:10.7717/peerj.10721)

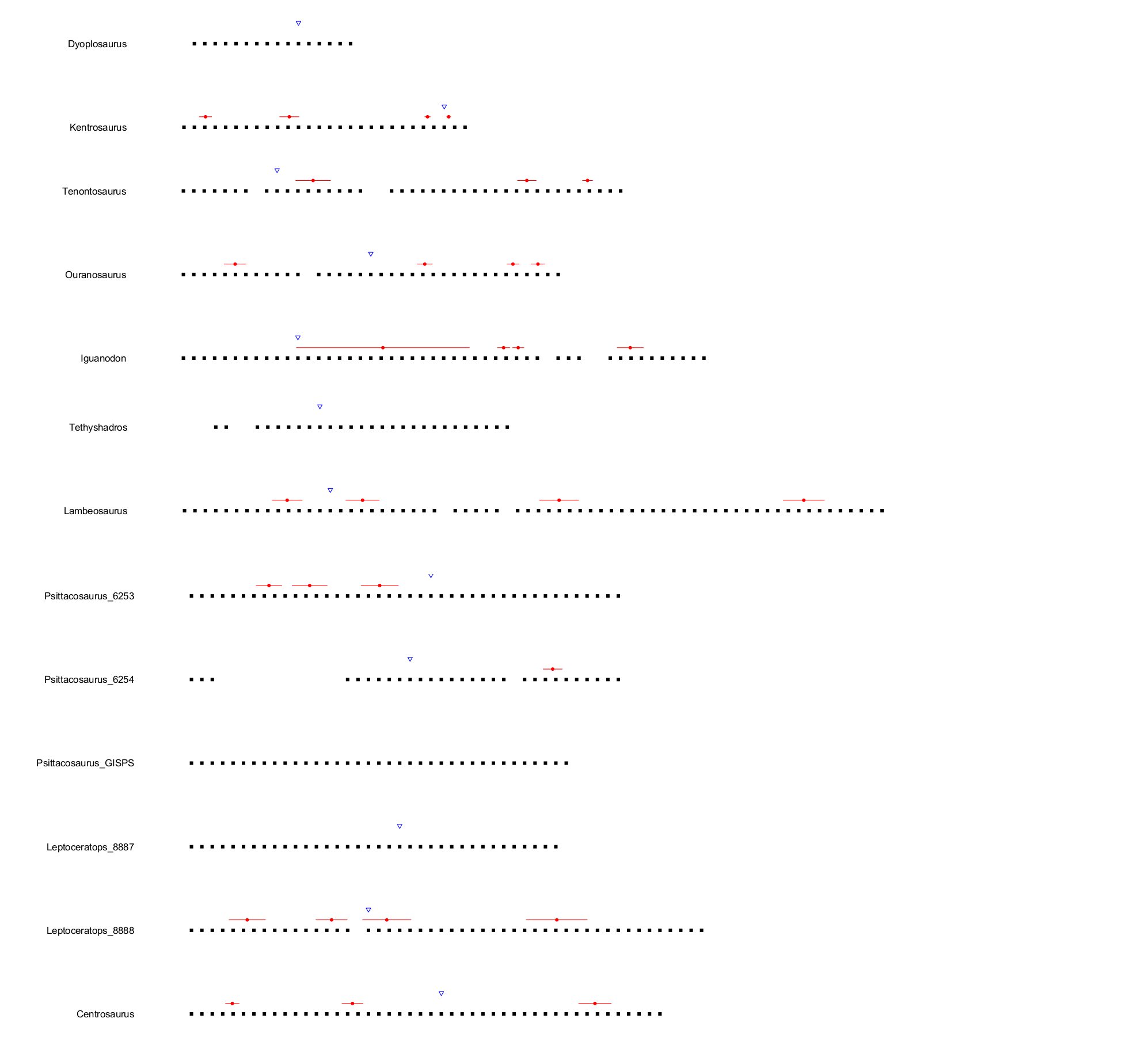

Supplement: Figure S1 — Aligned caudal centra (black squares, spaces indicate missing vertebrae), break points as calculated (red points with error bars) and transition point (where known, blue triangles) for all ornithischians in the study. [file peerj-09-10721-s002.jpg]

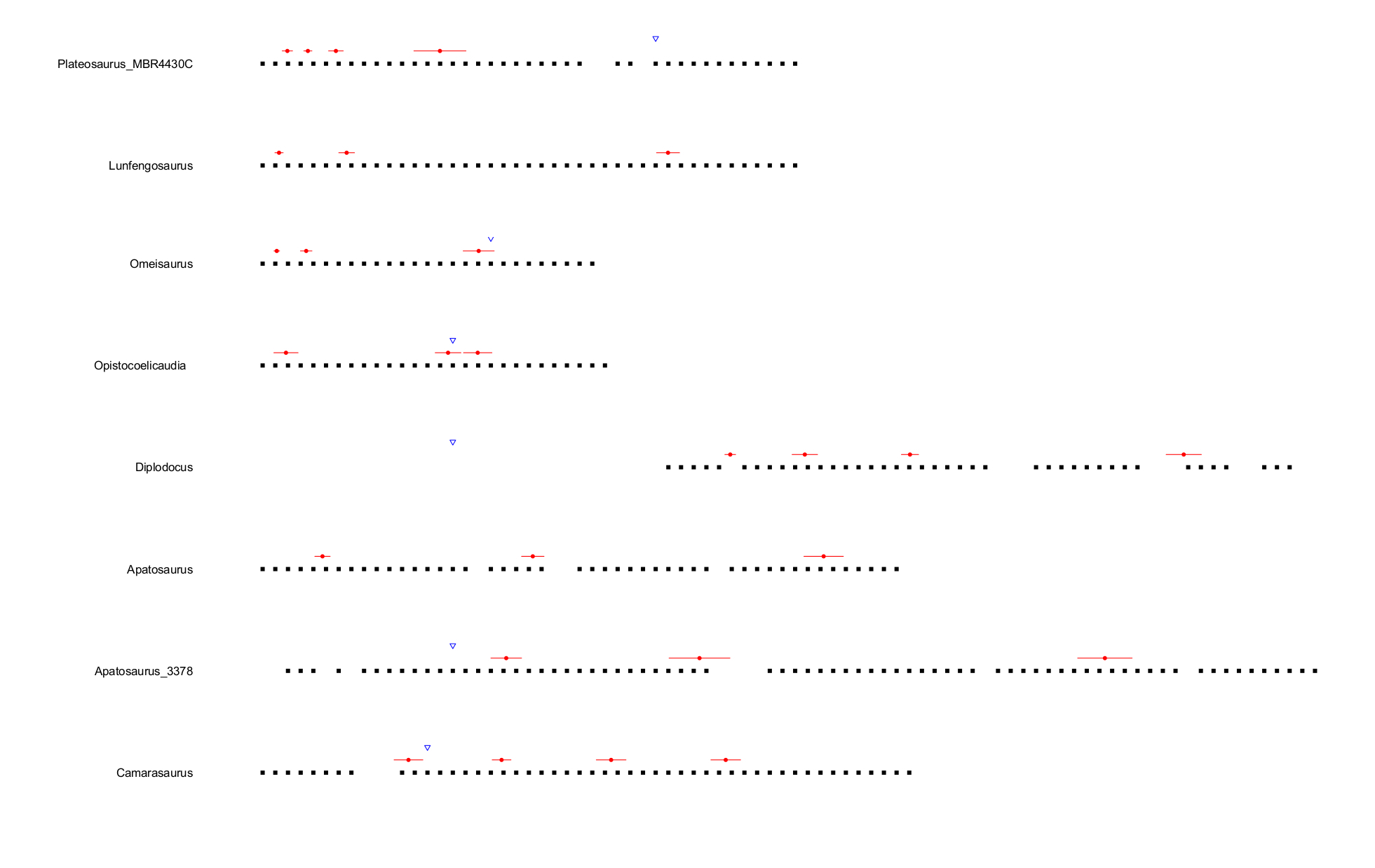

Supplement: Figure S2 — Aligned caudal centra, break points as calculated and transition point (where known) for all sauropodomorphs in the study. [file peerj-09-10721-s003.jpg]

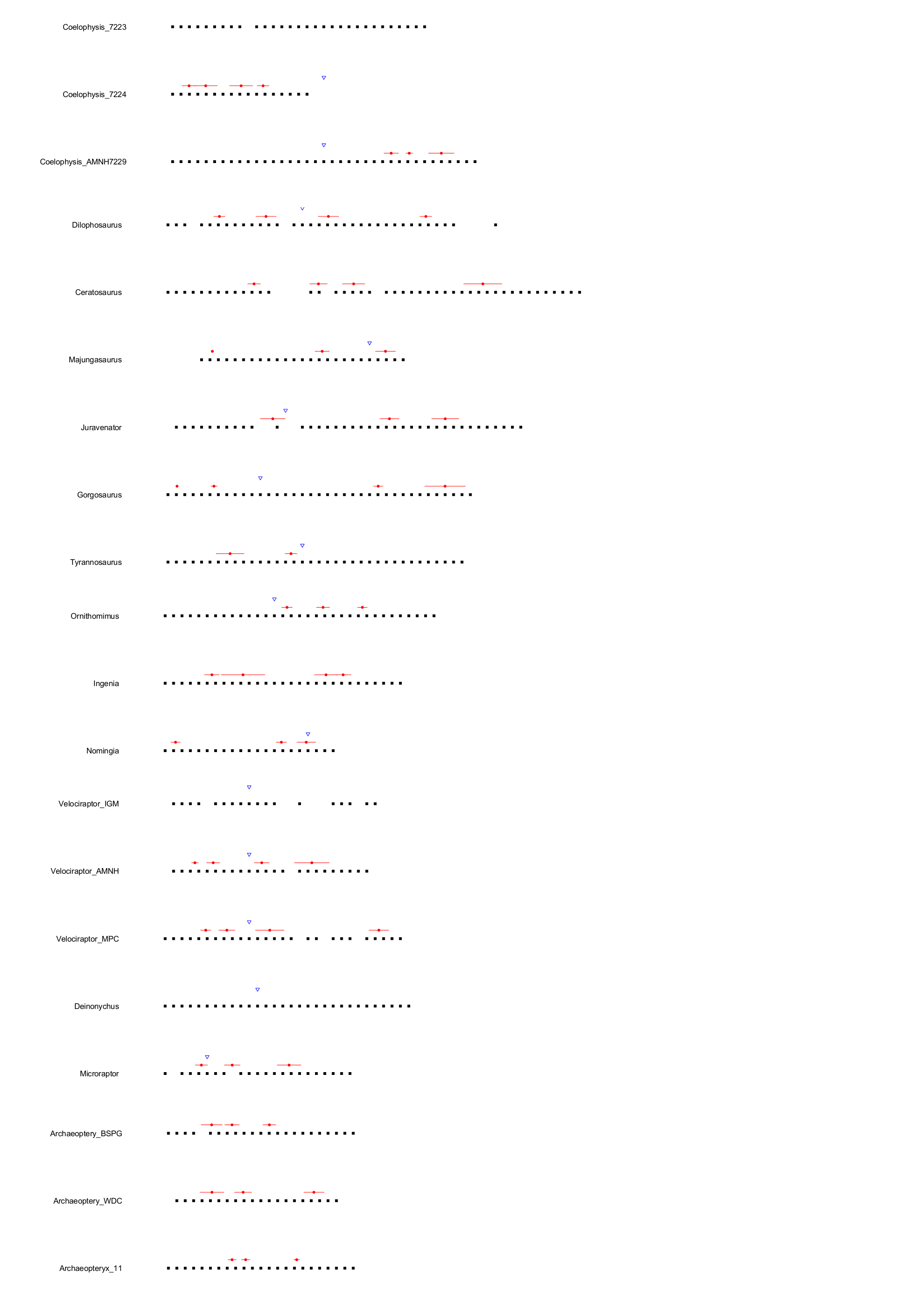

Supplement: Figure S3 — Aligned caudal centra, break points as calculated and transition point (where known) for all theropods in the study. [file peerj-09-10721-s004.jpg]

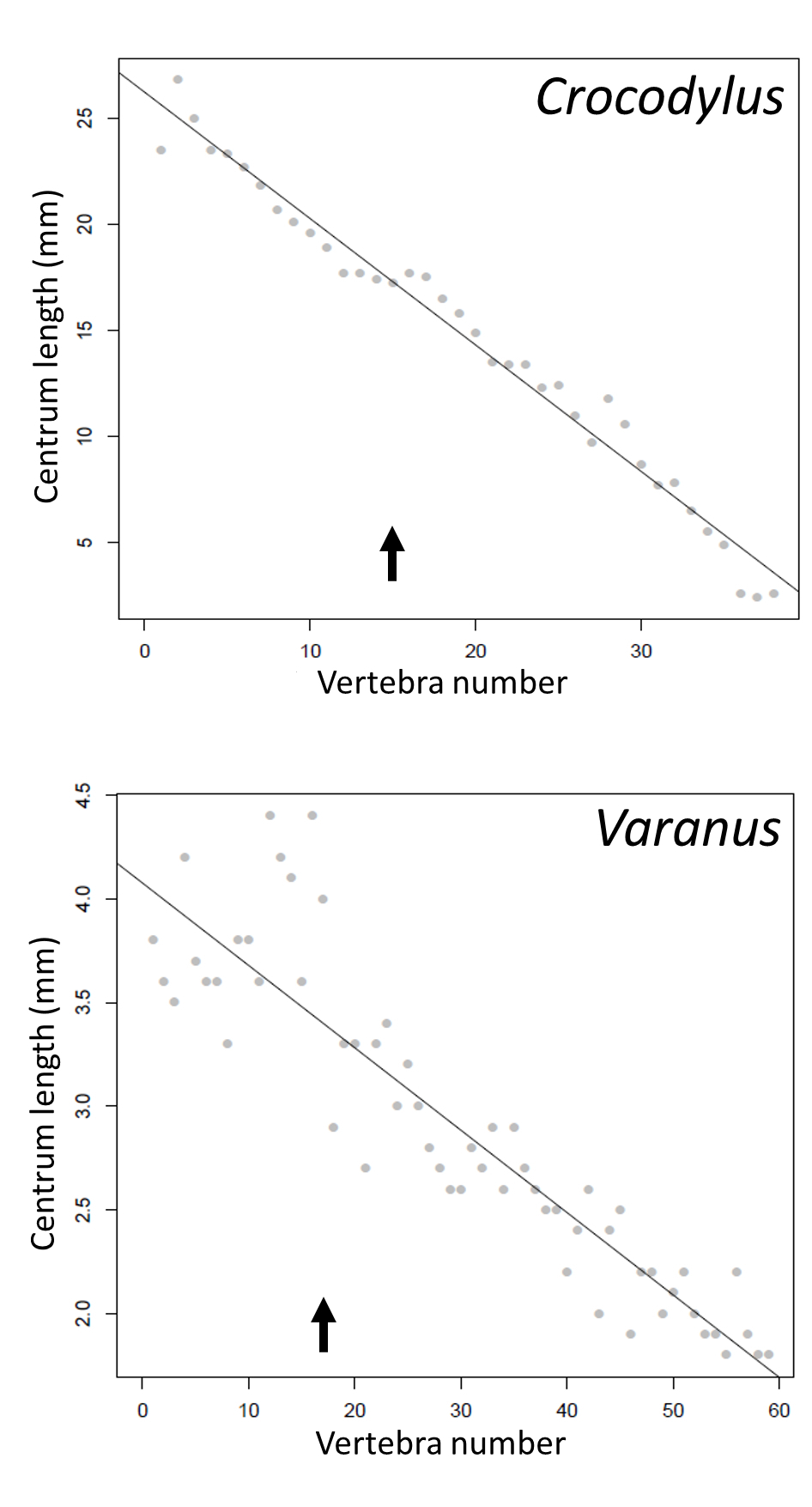

Supplement: Figure S4 — Centrum length patterns for Crocodylus niloticus and Varanus nilitocus. Both display a simpler pattern than that typical of the considered dinosaurs with a relatively consistent decrease in centrum length along the caudal series. The transition point (where known) is indicated by an arrow. [file peerj-09-10721-s005.png]
